# Oxidative Balance Score during Pregnancy Is Associated with Oxidative Stress in the CANDLE Study

**DOI:** 10.3390/nu14112327

**Published:** 2022-06-01

**Authors:** Frances A. Tylavsky, Luhang Han, Lauren M. Sims Taylor, W. Alex Mason, Kecia N. Carroll, Nicole R. Bush, Kaja Z. LeWinn, Melissa M. Melough, Terryl J. Hartman, Qi Zhao

**Affiliations:** 1 Department of Preventive Medicine, College of Medicine, University of Tennessee Health Science Center, Memphis, TN 38163, USA; ftylavsk@uthsc.edu (F.A.T.); lhan6@uthsc.edu (L.H.); lsims3@uthsc.edu (L.M.S.T.); wamason@uthsc.edu (W.A.M.); 2 Division of General Pediatrics, Icahn School of Medicine at Mount Sinai, New York, NY 10029, USA; kecia.carroll@mssm.edu; 3 Department of Psychiatry and Behavioral Health, Weill Institute for Neurosciences, University of California San Francisco, San Francisco, CA 94118, USA; nicole.bush@ucsf.edu (N.R.B.); kaja.lewinn@ucsf.edu (K.Z.L.); 4 Department of Pediatrics, University of California San Francisco, San Francisco, CA 94118, USA; 5 Center for Child Health, Behavior and Development, Seattle Children’s Research Institute, Seattle, WA 98121, USA; melissa.melough@seattlechildrens.org; 6 Department of Epidemiology, Rollins School of Public Health, Emory University, Atlanta, GA 303221, USA; terryl.johnson.hartman@emory.edu

**Keywords:** anti-oxidant balance, dietary intake, isoprostanes, lifestyle, oxidative stress, pregnancy

## Abstract

The objective of this study was to calculate an oxidative balance score (OBS) utilizing diet and lifestyle information collected from 1322 women during the second trimester of pregnancy in the Conditions Affecting Neurocognitive Development and Learning in Early Childhood study. An energy-adjusted OBS was calculated using nutrient information from a Food Frequency Questionnaire (FFQ), lifestyle measures, and plasma folate and vitamin D levels. Using the least absolute shrinkage and selection operator method, 91 food items from the FFQ were selected and they accounted for 82% of the variance in the OBS, with cruciferous vegetables, citrus fruits, fruit juice, and coffee being among the highest anti-oxidant predictors, and red meats and alcohol among the highest pro-oxidant contributors. Urinary F_2_-isoprostane, an objective indicator of oxidative stress, was lower with increasing OBS quintiles in a stairstep manner (*p* for trend = 0.0003), suggesting the possible utility of the OBS as an indicator of oxidative stress. The OBS was moderately correlated with the Healthy Eating Index (correlation coefficient = 0.6076), suggesting it provides a distinct measure of a healthy diet. In conclusion, the OBS may serve as a valid reflective indicator of urinary F_2_-isoprostanes and an epidemiological tool to inform intervention studies, in order to minimize oxidative stress during pregnancy.

## 1. Introduction

Oxidative stress is defined as an imbalance between the production and detoxification of oxidants. Oxidants are classified as ‘pro’, i.e., production of oxidation by products or as ‘anti’, i.e., ability to neutralize the oxidative products. Pro-oxidants include environmental factors such as exposure to radiation, air pollution, pesticides, industrial chemicals, and smoking cigarettes or other tobacco products. The consumption of certain foods, beverages, and nutrients may also have pro-oxidant effects, including exposure to air pollution, pesticides, and industrial chemicals, along with dietary factors such as alcohol, red meat, selected types of dietary fats (total fat, N-6 polyunsaturated fatty acids, and saturated fat), and iron. Common anti-oxidant nutrients and bioactive compounds include dietary N-3 polyunsaturated fatty acids, vitamins C and E, selenium, zinc, fiber, flavonoids, and glucosinolates and their respective dietary supplements. In addition, selected medications (e.g., aspirin and non-steroidal anti-inflammatory drugs (NSAIDS)) have anti-oxidant properties [1]. Oxidative products, known as reactive oxygen species (ROS), are generated as a byproduct of oxygen metabolism and adenosine triphosphate (ATP) production. Anti-oxidants neutralize or balance ROS primarily through scavenging of free radicals, chelating metal ions, regulating enzymes for host anti-oxidants, reducing enzymes involved with generating ROS, and modulating gut microbiota [1,2,3,4,5,6,7,8,9,10,11,12,13,14,15,16,17]. When ROS exceeds the anti-oxidant capacity in the body, increasing oxidative stress can cause tissue damage through ROS-induced apoptosis and necrosis [18] which may contribute to the development of chronic disease.

The measurement of urinary F_2_-isoprostanes is a valid and precise biomarker of oxidative stress [19,20], yet measurement is not always feasible due to the cost and availability of biospecimens. Thus, the calculation of an oxidative balance score (OBS) using the information on diet and lifestyle factors has been put forth to estimate oxidative stress using existing observational data. Higher OBS has previously been linked to the development of cancer, cardiovascular disease, diabetes, obesity, and cognitive function in aging populations [1]. The major challenge in understanding the association of OBS with health and development lies within the calculation of OBS. OBS characterizes individuals’ complex combined exposures to anti- and pro-oxidants based on nutrition and lifestyle behaviors. OBS has limitations, including the lack of standard calculations, the time frame with which the diet exposures were collected, and a lack of availability of all oxidants due to study-specific diet collection techniques, limiting the optimal evaluation of associations with the outcome of interest across studies.

Oxidative stress during pregnancy has been implicated in the pathophysiology of many reproductive complications, including miscarriage, preeclampsia, fetal growth restriction, and preterm birth [21,22]. The balance of pro- and anti-oxidants of the mother and the fetus affect the replication, differentiation, and maturation of the developing cells, which may also have long-term effects on the health outcomes of the children [22,23]. OBS, a comprehensive measure of oxidative balance, and indicative of life stressors, could be an important avenue for reducing negative outcomes in children. The objective of this paper was threefold; (1) to present the application of the OBS, utilizing diet and lifestyle information collected during the second trimester of pregnancy in the Conditions Affecting Neurocognitive Development and Learning in Early Childhood (CANDLE) study; (2) evaluate its validity with concurrent measurements of F_2_-isoprostanes [19]; (3) compare it with the Healthy Eating Index [24] and food consumption as a functional marker of diet selection as a basis for diet interventions, in order to optimize OBS status during pregnancy [25].

## 2. Materials and Methods

### 2.1. Study Subjects

All women included in this analysis were participants in the CANDLE study, a prospective pregnancy cohort of mother–child dyads in Shelby County, Tennessee (Memphis area) [26,27,28]. Briefly, 1503 healthy women aged 16–40 years and in their second trimester of a singleton pregnancy were enrolled between 2006 and 2011. Exclusion criteria included an existing chronic disease requiring medication (e.g., hypertension, diabetes, and sickle cell disease), known pregnancy complications (e.g., complete placenta previa and oligohydramnios), or plans to deliver at a nonparticipating hospital [27]. This study included 1322 CANDLE mothers with available maternal dietary data, of whom 990 were black (65%) and 437 were white (33%). The CANDLE study was conducted in accordance with the Helsinki Declaration and approved by the Institutional Review Board of the University of Tennessee Health Science Center. Informed consent was given by participants 18 years or older, while assent was given by those less than 18 years and consent was provided by their legally authorized representative prior to enrollment.

### 2.2. Maternal Measures

Self-administered questionnaires were used to collect sociodemographic information (age, race/ethnicity, education, insurance type, and marital status), lifestyle information (cigarette smoking and alcohol use during pregnancy), parity, and medical history at enrolment. Self-reported height and weight prior to pregnancy were collected at enrolment and used to calculate pre-pregnancy body mass index (pBMI) as weight (in kilograms) divided by the square of height (in meters), and participants were then categorized as underweight (<18.5 kg/m^2^), normal (18.5–24.9 kg/m^2^), overweight (25–29.9 kg/m^2^), or obese (>30 kg/m^2^).

### 2.3. Biomarker Measurement

Venous blood and spot urine were collected from mothers during baseline visits at 16 to 28 weeks gestation. Samples were transported on ice, centrifuged, aliquoted, and frozen at −20 °C within 6 h of collection. Plasma concentrations of 25-hydroxyvitamin D (25(OH)D), a stable [29,30] and reliable marker of vitamin D status [31], were measured using a commercial enzymatic immunoassay kit (Immunodiagnostic Systems, Tyne and Wear, UK) according to manufacturer’s instructions. Assays were performed at the University of Tennessee Health Science Center in a laboratory, which participates in the College of American Pathology Quality Assessment Program for 25(OH)D assays. NIST SRM972 Vitamin D was used for quality assurance of 25(OH)D. The minimum detection range of the assay was 2 ng/mL. The inter-assay variability was less than 6% for the laboratory assay controls, and precision was within 1 standard deviation (SD) of the mean vitamin D level. Plasma folate levels were assessed by a *Lactobacillus casei* microbiological assay at the University of Alabama at Birmingham [3]. The minimum detection limit of the assay was 3 ng/mL, with 4.8–5.9% intra-assay and 5.5–6.5% inter-assay variability for the laboratory assay controls. F2α isomer and 8-isoprostane (F_2_-isoprostanes) were measured in urine using mass spectrometry as previously described [32] with a precision of ±6% and an accuracy of 96%; F_2_-isoprostanes results were corrected for urinary creatinine (ng/mg-creatinine) to account for variable dilution.

### 2.4. Maternal Dietary Assessment during Pregnancy

The Block Food Frequency Questionnaire (FFQ) was administered by trained interviewers at enrollment during the second trimester (16–26 weeks of pregnancy) to assess participants’ usual intake of 111 food and beverage items and vitamin/mineral supplements during the previous three months. The Block FFQ has been shown to be a valid and reliable instrument to rank individuals according to dietary and nutrient intake [33]. Interviewers were trained by registered dietitians and re-certified by a registered dietitian based on a taped interview every six months. The FFQ was processed by NutritionQuest (Berkeley, CA, USA) to assess macro and micronutrient intake. The 111 food/beverage items were categorized into 39 pre-defined food groups that were based on similarities in nutrient composition, culinary, or consumption habits. An overview of these predefined food groups and the corresponding food items derived from the FFQ are shown in Supplemental Table S1.

### 2.5. Oxidative Balance Score Calculation

Pro- and anti-oxidant contributors were selected based on those available from the report of food intake and nutrients obtained from the FFQ, plasma folate, plasma 25(OH)D, and lifestyle variables identified as pro- or anti-oxidants from previous research [1]. Supplemental Table S2 contains the pro- and anti-oxidants selected for this study and the scientific rationale for inclusion. Complete data were available for all the anti-oxidants and pro-oxidants except for the plasma 25(OH)D, mothers taking NSAIDs at enrollment, and pBMI, for which there were less than 2.4% entries missing, and were thus imputed in SAS 9.4 using the MI procedure. We coded the binary variables NSAID use and smoking status as 1 for use/smoking and 0 for nonuse/nonsmoking. For continuous nutrient/food factors, regression residuals after adjusting for daily total energy intake were calculated using linear regression models. The *z*-scores of residuals were calculated ((X minus population mean)/population standard deviation). Pro-oxidant *z*-scores were multiplied by −1 and anti-oxidant *z*-scores were multiplied by 1 and summed for a final OBS. A higher OBS were considered as being favorable, indicating lower levels of pro-oxidants but higher levels of anti-oxidants.

### 2.6. Statistical Analysis

To identify food intake associated with the OBS based on the 111 food items from the FFQ, the least absolute shrinkage and selection operator (LASSO) technique was implemented [34] using the R package “glmnet”. The LASSO performs variable selection by forcing some of the coefficient estimates to zero when the tuning parameter λ is sufficiently large and selecting for non-zero predictors. In this study, data were randomly split into the training set (70% of the sample) and the test set (30% of the sample). In the training set, a ten-fold cross-validation applied to the LASSO fits to select the λ that minimizes training mean square error (MSE). We then evaluated the model performance in the independent test set and used the test MSE as the criteria. The final models selected the most predictive food items in association with OBS. To access associations between the OBS and F_2_-isoprostanes during pregnancy, we used linear regression models by taking the OBS as a continuous variable. Potential confounding factors, such as maternal demographic (age, race, and marital status), socioeconomic factors (education, insurance type and adjusted income), and reproductive history (parity), were also included in the linear regression models. The Healthy Eating Index—2010 was calculated based on the 2010 Dietary Guidelines for Americans [35]. All analyses were performed using R Statistical Software (version 4.0.2, R Core Team, Vienna, Austria, https://www.R-project.org/).

## 3. Results

### 3.1. Participant Characteristics

At enrollment in the study, the average age of the mothers was 26.3 years. Over half identified as black, reported completing high school or less, were enrolled in Medicaid/Medicare health insurance, were overweight or obese prior to pregnancy, reported cohabitation, and were primiparous (Table 1). Participants who were enrolled in CANDLE but were excluded from this analysis were similar in sociodemographic characteristics to those included in the analysis (Supplemental Table S3).

### 3.2. Oxidative Balance Score

The OBS ranged from −40.8 to 64.9, with an average (SD) of 37.9 (17.5). In general, maternal age and education were higher with increasing OBS quintiles, and fewer mothers identified as black, reported use of public insurance, or were overweight/obese was higher (*p* < 0.0001 for linear trend tests) (Table 1). The majority of the energy-adjusted anti-oxidant components of the OBS were higher with increasing quintiles and the energy-adjusted pro-oxidant components of the OBS were lower with increasing quintiles (*p* < 0.0001 for linear trend tests) (Table 1), except for omega-3 fatty acids, the use of NSAIDS, alcohol consumption, and smoking (*p* = 0.0616, 0.0375, 0.4215, and 0.0180 for linear trend tests, respectively). HEI scores increased with quintiles of OBS, indicating a higher quality diet associated with higher OBS (*p* < 0.0001 for linear trend test) (Table 1). The OBS was moderately correlated with the Healthy Eating Index (correlation coefficient = 0.6076), suggesting it provides a distinct measure of a healthy diet.

### 3.3. Food Sources Contributing to OBS

The LASSO technique selected 91 food items from the FFQ that accounted for 82% of the variance in the OBS (Supplementary Table S4).

The top food items contributing to increased OBS were from the following food groups: higher OBS comprise including coffee, tea, cruciferous and yellow vegetables, dairy, seafood, organ meats, pasta, green salad, power bars, and tofu. The foods that contributed most to a decreased OBS were from the red meat and alcohol groups (Table 2). Due to the comprehensive nature of the OBS, each food item was associated with minor increases/decreases to the OBS, as reflected in the listed beta coefficients, yet each provides evidence on which foods could be considered for possible intervention to optimize the OBS.

### 3.4. Association between Oxidative Balance Score and F_2_-Isoprostanes

In a test of validity using those with isoprostane concentrations (*n* = 1019), the associations between the OBS and F_2_-isoprostane levels are presented in Table 3. F_2_-isoprostane levels were lower, indicating a lower systemic oxidative stress with increasing OBS quintiles. Using quintile one (lowest) as a reference, F_2_-isoprostane levels showed a significant decreasing trend across quintiles of OBS (*p* value = 0.0003). After adjusting for sociodemographic and reproductive history, the associations between F_2_-isoprostanes and OBS were slightly attenuated but remained statistically significant.

## 4. Discussion

In the CANDLE Study, a well characterized, large and socioeconomically and racially diverse U.S. cohort, the nutrient-, nutrient biomarker-, and lifestyle-generated OBS was inversely associated with F_2_-isoprostanes, providing that empirical support for its use as an indicator of oxidative stress balance. Dietary components associated with lower urinary F_2_-isoprostane concentrations included fruits and their juices, cruciferous vegetables, pizza, and hot tea, while the consumption of bread products, sweets and desserts were associated with higher oxidative stress. These findings established a foundation for future development of effective intervention methods for reducing oxidative stress during pregnancy. The positive association between OBS and HEI suggests that our calculation captures not only oxidative balance but also provides an estimate of a healthy diet, thus the components of HEI might be used as a basis for proposing diet interventions to reduce oxidative stress.

### 4.1. Biomarker Measures of Oxidative Stress

Biomarkers of oxidative stress are reflective of an impairment of anti-oxidant enzymes and non-enzymatic networks that may lead to imbalances between the production and detoxification of oxidants. Oxidative stress biomarkers include oxidized DNA, malondialdehyde, oxidized low-density lipoprotein, glutathione, advanced glycation end products, nitrotyrosine, protein carbonyls, and F_2_-isoprostanes [36]. Many of these oxidative stress biomarkers have been associated with pregnancy complications including pre-eclampsia, gestational-diabetes mellitus, preterm birth, and intrauterine growth restriction [36], which are known predictors for acute and chronic health problems for the mother. In addition, these biomarkers have been associated with fetal loss or dysregulation of infant physiology leading to a risk of long term chronic disease or developmental disorders in the offspring such as cardiovascular disease [37], diabetes mellitus [38], and hypertension [39], indicating their crucial influence in the development of lifestyle diseases. The oxidative stress markers can be specific to certain anti-oxidant enzymes or non-enzyme pathways or used as an overall measure of oxidative stress. The biomarker F_2_-isoprostane, which is considered as the most potent isoprostane biomarker, reflects the oxidative status of urine [40] and is considered a gold standard measurement of oxidative stress in physiological and pathophysiological states [41]. For this investigation, the urinary F2α isomer 8-isoprostane was used to estimate overall oxidative stress and cannot be linked to a specific pathway. In our relative validity analyses, the inverse association between OBS and F_2_-isoprostanes indicates that for the CANDLE cohort, OBS may be a reasonable surrogate of oxidative balance and can act as a possible predictor, mediator, or confounder in future investigations.

### 4.2. Diet/Lifestyle A Measure of Oxidative Stress

Recognizing the limitations and cost of measuring individual anti- and pro-oxidants, the idea of measuring the total anti-oxidant capacity (TAC) of foods and biological fluids was readily embraced because it could integrate the individual anti-oxidant action of different compounds and their additive, synergistic, or antagonistic interactions. Unfortunately, due to the chemical diversity of pro- and anti-oxidants in foods and plasma, large inter-lab variabilities in measurements, limitations of food databases, and a lack of concurrent validity with biological measures, TAC estimates have not been clearly or consistently associated with the development, onset, and progression of non-communicable disease [42]. In contrast, OBS do not require access to specific food-related databases, and it additionally considers non-dietary factors such as smoking, selected medication use, and weight status. OBS has been consistently associated with biological measures of oxidative stress in the current investigation and other studies [43,44] and has been positively associated with mortality and morbidity in some, but not all, chronic diseases [1]. Although it is beyond the scope of this paper to evaluate the association of OBS with morbidity in this cohort, this study is the first to use OBS from diet during pregnancy with a potential to link it to health outcomes associated with pregnancy and the developmental origins of health and disease in early and middle childhood. Limitations of most OBS research include a lack of comprehensive food, nutrient, and lifestyle variables to incorporate into a robust OBS calculation. Our study included nutrients, lifestyle factors, and nutrient biomarkers, resulting in a comprehensive calculation to characterize OBS. Our results showed that all variables used in the OBS calculation appropriately increased or decreased with increasing quintiles of OBS for pro- and anti-oxidants, suggesting the relative contribution of a wide variety of nutrients/lifestyle variables to fully estimate OBS. We identified a robust association of OBS with F_2_-isoprostanes, which is a reasonable estimate for oxidative stress and discriminates across OBS quintiles. Additionally, our investigation showed a robust association between the OBS and the HEI and identified food items that promote a high anti-oxidant intake. All these findings provide evidence that OBS may be a useful measure of diet that could be used as a basis for investigation of the health outcomes associated with pregnancy, the developmental origins of health and disease, as well as possible recommendations for changes in diet to counteract the negative effects of oxidative stress in maintaining health and mitigating disease.

### 4.3. Sources of Oxidants

Natural anti-oxidants such as ascorbic acid, α-tocopherol, carotenoids, minerals, and polyphenols are from commonly consumed fruits, vegetables, beverages, cereals, and other food products. Across the quintiles of OBS, incremental increases in food-based anti-oxidants and decreases in pro-oxidants were observed, indicating the inclusion of selected nutrients contributed as expected to the OBS calculation. A summary score captures the total balance of the diet but does not reflect nutrient–nutrient/food interactions as a consequence of personal metabolism and genetic milieu. The association of OBS with the food groups rich in fruits and vegetables and hot tea confirm that food selection, as predicted by foods rich in anti-oxidants, provides a basis for further investigative efforts or evaluation for potential interventions to counteract the detriments of oxidative stress, such as seen with N-3 PUFAs [45]. As would be predicted, diets characterized by sweets and refined carbohydrates are indicative of lower anti-oxidant intake and, thus, less oxidative-stress-buffering capacity. Clearly, oxidative stress research using diet is in its early stages, but having diet-based OBS as an estimate of oxidative balance opens up a plethora of investigations regarding the development of disease.

### 4.4. Strengths and Limitations

This study leveraged a large, diverse cohort with a comprehensive panel of pro- and anti-oxidant factors from diet, lifestyle, and biomarkers, in order to calculate an OBS as an indicator of oxidative stress for future association studies. The OBS provides a comprehensive evaluation of oxidative stress by incorporating both pro- and anti-oxidants. This study provides a first report of lifestyle-based OBS in a large diverse population in the second trimester of pregnancy, characterized by a high degree of low socioeconomic status. The finding extends OBS research to include food-group-based recommendations into strategies to increase the oxidative balance throughout one’s diet, in order to decrease oxidative stress. Using the robust LASSO technique our analyses identified 91 food items that accounted for 82% of the variance of the OBS calculation, underscoring the need to use a comprehensive diet measure that reflects usual intake such as a FFQ used in this study. This research is not without limitations. The OBS used in this study was limited to 24 nutrients, 4 lifestyle variables, and 2 nutrient biomarkers, thus, it may not be as robust with the inclusion of additional biomarkers. Chronic inflammation is integrally related to free radical generation and oxidative stress [46], but this investigation did not have access to biomarkers of inflammation for evaluation. This study presents summary results that could be used to inform future epidemiological studies, yet the assessment of specific metabolic pathway markers for oxidative stress may be beneficial for addressing certain targeted research questions. Another limitation is that only one-time FFQ was collected and this may not be sufficient to evaluate dietary intake during the entire pregnancy.

## 5. Conclusions

In this large, well-characterized, and socioeconomically and racially diverse U.S. cohort, we calculated a measure of oxidative balance using diet, lifestyle, and biomarkers during pregnancy for use in future association studies evaluating pregnancy outcomes, neurodevelopmental outcomes in childhood, and the developmental origins of health and disease. In pregnant women, the OBS was associated with F2-isoprostanes, a measure of oxidative stress. All anti- and pro-oxidants used to calculate the OBS increased/decreased as expected across the increasing quintiles of OBS. As expected, the food groups most predictive of OBS were those highest in anti-oxidants (e.g., fruits and vegetables) and those with low anti-oxidant content (e.g., red meats, sweets, desserts, and alcohol), which are helpful in planning intervention studies to minimize oxidative stress. The magnitudes of the effect for individual anti- or pro-oxidants were minimal, suggesting that the totality of an individual’s diet/lifestyle is important for modulating oxidative stress and its sequelae. Future studies are needed to sharpen the science of OBS as an exposure for pregnancy outcomes and child development, its role in the development of chronic disease from early to later childhood, and to assess the role of potential protective effect modifiers.

## Figures and Tables

**Table 1:** Characteristics according to Oxidative Balance Score, by Quintiles presented as mean (SD) or *n* (%).

Characteristic	All Mean (SD)/*n* (%)	Q1 [−40.8, −9.2] Mean (SD)/*n* (%)	Q2 [−9.2, −3.7] Mean (SD)/*n* (%)	Q3 [−3.7, 1.6] Mean (SD)/*n* (%)	Q4 [1.6, 8.4] Mean (SD)/*n* (%)	Q5 [8.4, 64.9] Mean (SD)/*n* (%)	*p* Value ^4^
*n*	1322	265	264	264	264	264	
**Maternal**							
Age, years, mean/SD	26.3 (5.4)	24.0 (0.3)	25.9 (0.3)	26.6 (0.3)	27.2 (0.3)	27.7 (0.3)	<0.0001
Black, *n*/%	847 (64.1%)	241 (90.9%)	197 (74.6%)	149 (56.4%)	140 (53.0%)	120 (45.3%)	<0.0001
Education (≤12 years), *n*/%	761 (57.6%)	208 (78.5%)	178 (67.4%)	129 (48.9%)	127 (48.1%)	119 (44.9%)	<0.0001
Marital status (single), *n*/%	545 (41.2%)	158 (59.6%)	125 (47.3%)	98 (37.1%)	87 (33.0%)	77 (29.1%)	<0.0001
Insurance (Medicaid or Medicare), *n*/%	755 (57.1%)	216 (81.5%)	173 (65.5%)	133 (50.4%)	115 (43.6%)	118 (44.5%)	<0.0001
Parity (primiparous), *n*/%	543 (41.1%)	81 (30.6%)	113 (42.8%)	123 (46.6%)	114 (43.2%)	112 (42.3%)	0.0128
Overweight/Obese, *n*/%	701 (53.0%)	160 (60.4%)	165 (62.5%)	129 (48.9%)	133 (50.4%)	114 (43.0%)	<0.0001
**Oxidative Balance Score Components ^1^**							
**Total energy intake, kcals**	2728.9 (1678.0)	3201.2 (115.4)	2596.6 (98.9)	2377.0 (76.8)	2369.7 (90.5)	3096.9 (117.4)	0.1823
**Anti-oxidants**							
Alpha-tocopherol, mg ^1,2^	3.85 (1.07)	3.34 (0.04)	3.45 (0.04)	3.71 (0.05)	4.04 (0.05)	4.69 (0.09)	<0.0001
Vitamin C, mg ^1,2^	72.2 (31.1)	56.1 (1.5)	62.5 (1.5)	69.5 (1.7)	81.3 (2.0)	91.4 (2.0)	<0.0001
Vitamin D, IU	77.5 (50.3)	58.0 (2.2)	68.0 (2.5)	76.9 (2.8)	89.7 (3.2)	95.0 (3.9)	<0.0001
Niacin, mg ^1,2^	10.7 (2.3)	9.5 (0.1)	10.3 (0.1)	10.5 (0.1)	11.0 (0.1)	11.9 (0.2)	<0.0001
Riboflavin, mg ^1,2^	1.01 (0.24)	0.87 (0.01)	0.94 (0.01)	1.00 (0.01)	1.09 (0.02)	1.18 (0.02)	<0.0001
Dietary Folate Equivalents, mcg ^1,2^	282.8 (92.2)	225.3 (3.7)	251.5 (3.8)	275.6 (4.7)	316.0 (5.9)	345.5 (6.4)	<0.0001
Vitamin K, mcg ^1,2^	99.6 (74.9)	57.6 (2.4)	69.5 (2.5)	85.8 (3.2)	125.6 (4.9)	159.1 (5.8)	<0.0001
Alpha-carotene, mcg	195.2 (233.9)	91.1 (6.5)	124.2 (7.9)	165.4 (9.4)	226.7 (13.9)	368.3 (21.7)	<0.0001
Beta-carotene, mcg	1972.5 (1411.1)	1060.4 (48.2)	1340.5 (48.2)	1705.7 (58.6)	2428.5 (78.9)	3325.7 (103.6)	<0.0001
Cryptoxanthin, beta, mcg	106.2 (67.1)	73.2 (2.9)	88.9 (3.2)	105.5 (3.8)	125.0 (4.2)	138.3 (4.8)	<0.0001
Lutein-Zeaxanthin, mcg	1793.4 (1422.9)	984.7 (44.6)	1219.5 (43.7)	1517.0 (55.5)	2290.4 (92.5)	2954.2 (112.9)	<0.0001
Lycopene, mcg	2590.8 (1850.5)	1902.6 (78.9)	2251.0 (71.7)	2547.6 (105.5)	2709.8 (109.3)	3542.0 (157.2)	<0.0001
Retinol, mcg	243.1 (100.6)	211.8 (4.3)	228.0 (5.0)	239.4 (5.6)	262.3 (6.4)	274.1 (8.1)	<0.0001
Omega-3 fatty acids, g ^1^	0.82 (0.22)	0.84 (0.01)	0.83 (0.01)	0.82 (0.01)	0.81 (0.01)	0.81 (0.01)	0.0616
Dietary Fiber, g	8.9 (3.0)	6.7 (0.1)	7.7 (0.1)	8.7 (0.1)	10.1 (0.2)	11.5 (0.2)	<0.0001
Glutathione, g	22.2 (5.6)	19.3 (0.3)	20.9 (0.3)	21.9 (0.3)	23.7 (0.3)	25.1 (0.4)	<0.0001
Total Flavonoid, mg ^3^	154.4 (112.0)	111.4 (5.0)	130.7 (5.7)	152.7 (6.0)	190.1 (8.3)	187.1 (7.6)	<0.0001
Copper, mg ^1,2^	0.65 (0.15)	0.53 (0.01)	0.59 (0.01)	0.63 (0.01)	0.70 (0.01)	0.78 (0.01)	<0.0001
Magnesium, mg ^1,2^	142.9 (35.5)	112.1 (1.3)	127.5 (1.4)	138.8 (1.4)	159.1 (1.9)	177.1 (2.2)	<0.0001
Selenium, mg ^1,2^	48.4 (8.7)	45.7 (0.5)	47.7 (0.5)	48.3 (0.5)	49.1 (0.5)	51.0 (0.5)	<0.0001
Zinc, mg ^1,2^	5.6 (1.3)	5.1 (0.1)	5.2 (0.1)	5.5 (0.1)	5.7 (0.1)	6.2 (0.1)	<0.0001
Plasma Vitamin D	22.3 (8.5)	18.0 (0.4)	21.5 (0.5)	23.6 (0.5)	23.8 (0.5)	24.5 (0.6)	<0.0001
Plasma Folate	23.8 (12.9)	16.6 (0.5)	22.4 (0.7)	26.5 (0.8)	25.9 (0.8)	27.8 (0.9)	<0.0001
Caffeine, mg	18.3 (22.2)	14.1 (1.0)	16.1 (1.3)	17.5 (1.3)	21.5 (1.5)	22.0 (1.7)	<0.0001
NSAIDS, yes/no	23 (1.7%)	0 (0.0%)	6 (2.3%)	5 (1.9%)	4 (1.5%)	8 (3.0%)	0.0375
**Pro-oxidants**							
Total Fat, g	40.6 (5.6)	43.5 (0.3)	41.8 (0.3)	40.5 (0.3)	38.7 (0.3)	38.3 (0.3)	<0.0001
Saturated fatty acid, g	13.2 (2.4)	14.5 (0.1)	13.8 (0.1)	13.2 (0.1)	12.5 (0.1)	12.1 (0.1)	<0.0001
Omega-6 fatty acids, g ^1^	7.3 (1.5)	7.6 (0.1)	7.5 (0.1)	7.3 (0.1)	7.2 (0.1)	7.1 (0.1)	<0.0001
Iron, mg ^1,2^	7.4 (1.7)	6.5 (0.1)	6.9 (0.1)	7.3 (0.1)	7.8 (0.1)	8.5 (0.1)	<0.0001
Alcohol Intake, g	0.03 (0.15)	0.04 (0.02)	0.02 (0.01)	0.02 (0.00)	0.02 (0.00)	0.03 (0.01)	0.4215
Pre-pregnancy BMI, kg/m^2^	27.4 (7.4)	28.8 (0.5)	28.6 (0.4)	26.9 (0.5)	26.9 (0.4)	26.0 (0.4)	<0.0001
Smoking during pregnancy, *n*/%	86 (6.5%)	14 (5.3%)	18 (6.8%)	15 (5.7%)	15 (5.7%)	24 (9.1%)	0.1800
**Dietary Index**							
HEI total score	60.2 (11.4)	49.4 (0.57)	55.6 (0.54)	60.8 (0.51)	65.7 (0.55)	69.6 (0.54)	<0.0001

^1^ Energy Adjusted; mean/SD; ^2^ Total intake from food plus supplements; ^3^ Sum of isoflavone daidzein, isoflavone genistein, isoflavone glycitein, anthocyanidin cyanidin, anthocyanidin petunidin, anthocyanidin delphinidin, anthocyanidin malvidin, anthocyanidin pelargonidin, anthocyanidin peonidin, flavan-3-ol catechin, flavan-3-ol epigallocatechin, flavan-3-ol epicatechin, flavan-3-ol epicatechin 3-gallate, flavan-3-ol epigallocatechin 3-gallate, flavan-3-ol theaflavin, flavan-3-ol thearubigin, flavanone eriodictuol, flavanone hesperetin, flavanone naringenin, flavone apigenin, flavone luteolin, flavonol isorhamnetin, flavonol kaempferol, flavonol myricetin, flavonol quercetin, flavan-3-ol theaflavin 3,3′-digallate, flavan-3-ol theaflavin 3′-gallate, flavan-3-ol theaflavin 3-gallate, flavan-3-ol gallocatechin, proanthocyanidins monomers, proanthocyanidins dimers, proanthocyanidins trimers, proanthocyanidins 4–6 mers, proanthocyanidins 7–10 mers, proanthocyanidins polymers (>10), mg. ^4^ Linear trend tests.

**Table 2:** Top food item contributors to the oxidative balance score.

Food Item	Food Group	Beta ^1^
Coffee	Coffee	0.16
Spinach	Cruciferous vegetable	0.49
Broccoli	Cruciferous vegetable	0.17
Slimfast	Dairy	0.17
Oysters	Fish and other seafood	0.76
Shellfish	Fish and other seafood	0.30
Liver	Organ meats	1.08
Spaghetti with meat sauce	Rice, pasta, mixed, dishes	0.19
Green salad	Salad dressing	0.21
Power bars	Snacks	0.20
Hot tea	Teas	0.23
Tofu	Tofu and meat substitutes	0.65
Carrots	Yellow vegetables	0.39
Sweet potato	Yellow vegetables	0.31
Ribs	Red meat	−0.35
Liquor	Alcohol	−7.31

^1^ Change in oxidative balance score per 1 unit increase in the serving of the food item.

**Table 3:** Associations between oxidative balance score (OBS) during pregnancy and F2-isoprostanes (*n* = 1019).

OBS *z*-Score	F_2-_Isoprostanes				
		Unadjusted		Adjusted ^1^	
	Mean (SE)	Beta	*p* Value ^2^	Beta	*p* Value ^2^
Continuous Score		−0.01	0.001	−0.01	0.006
Reference (quintile 1)	2.54 (0.08)	--	--	--	--
Quintile 2	2.34 (0.07)	−0.19	0.063	−0.18	0.085
Quintile 3	2.22 (0.07)	−0.32	0.003	−0.30	0.006
Quintile 4	2.21 (0.07)	−0.33	0.002	−0.29	0.010
Quintile 5	2.18 (0.08)	−0.36	0.001	−0.35	0.002

^1^ Adjusted for maternal demographic (age, race, and marital status), socioeconomic (education, insurance type, and adjusted income), and reproductive history (parity) characteristics. ^2^ Quintile 1 is the reference group for comparisons.

## Data Availability

The data presented in this study are available upon request and approval by the CANDLE study.

## Supplementary Materials

The following supporting information can be downloaded at: https://www.mdpi.com/article/10.3390/nu14112327/s1, Table S1: 111 Food Frequency Food items placed within 39 food Groups; Table S2: Selection of pro- and anti-oxidants for developing the oxidative balance score (OBS); Table S3: Socioeconomic characteristics of the study population; Table S4: Food Items from Food Frequency Questionnaire associated with Oxidative Balance Score (R^2^ = 0.82).
