# Supplementary material for: Oxidative Balance Score during Pregnancy Is Associated with Oxidative Stress in the CANDLE Study
Source: Nutrients. 2022 Jun 1;14(11):2327. doi: 10.3390/nu14112327 (PMC9182929; doi:10.3390/nu14112327)
Supplement: Supplementary file 1 [file nutrients-14-02327-s001.zip › nutrients-1728411-supplementary.pdf]

Table S1: 111 Food Frequency Food items placed within 39 food Groups.

Food groups	Food items
Red meat	Beef, pork, ribs, veal, meat loaf, and hamburger (cheeseburger), meatballs, meat sandwiches
Processed meat	Breakfast sausage, chorizo, Polish sausage, Italian sausage, bacon, hot dogs, and lunch meats
Organ meat	Liver, pig feet, neck bones and menudo
Poultry	Roasted chicken or turkey
Mixed meat	Tacos, burritos, enchiladas, tamales, other mixed beef and chicken dishes
Fish and other seafood	Oysters, shellfish, tuna (salad, casserole, etc.) and other fish
Fried chicken and fish	Fried chicken, chicken nuggets, wings, patties and fried fish or fish sandwich
Egg	Breakfast egg sandwiches and other eggs
Dairy	Milk, milk on cereal, yogurt, cheese, ice cream, and Slim Fast
Sugar-sweetened beverages	Hi-C, drinks with some juice, Kool-Aid or lemonade or sports drinks like Gatorade or fruit flavored drinks (not including iced teas), and soft drink, Iced Tea
Fruit juices	Real orange juice, tomato juice or V-8, apple, grape, pineapple juice, or fruit smoothies
Alcohol	Beer and liquor
Wine	Wine
Coffee	Coffee
Water	Water
Other fruit	Bananas, apples or pears, peaches, cantaloupes, watermelons, and other fresh fruits
Citrus fruits	Oranges, tangerines, grapefruits, strawberries
Canned fruit	Canned fruit
Tomatoes	Tomatoes and tomato or V-8 juice
Yellow vegetables	Carrots and sweet potato
Cruciferious vegetable	Broccoli, coleslaw, spinach, greens
Potatoes	Potatoes (not fried), mashed, boiled, baked, or potato salad
French fries	French fries
Other vegetables	Corn, green beans, vegetable stew, vegetable soup, and other vegetables
Legumes	Refried beans, bean, lentil or split pea soup, and other beans
Tofu and meat substitutes	Tofu and meat substitutes
Mayonnaise, margarine and butter	Margarine, butter and mayonnaise
Salad dressing	salad dressing
Condiments	Jelly, catsup, and mustard
Whole grains	Cooked cereal such as grits, oatmeal or cream of wheat
Cold breakfast cereal	Cold cereal
Bread products	Pancakes, biscuits, croissants, rolls, bagels, tortillas (flour), corn bread or muffins, hush puppies, white, whole wheat or dark, bread
Pizza	Pizza
Rice, pasta, mixed and dishes	Spaghetti with meat sauce, macaroni and cheese, rice, other soup, and other noodles
Nuts	Nuts, seeds and peanut butter

Sweets and desserts	Donuts, cakes, cookies, pumpkin pie, other pie, and other candy
Snacks	Power bars, breakfast bars, chips, and crackers
Teas	Hot teas
Chocolates	Chocolate syrup or sauce, chocolate candy

Table S2: Selection of pro- and antioxidants for developing the oxidative balance score (OBS).

Variable	Role	Use in Previous OBS Research	Rationale for the inclusion of some components in a priori OBS in relation to oxidative stress.
**Variables from Food Frequency Questionnaire**			
Food energy	Confounder	Yes	Used to adjust nutrient and reported intake
Alpha-carotene	Anti	Yes	Deactivators of singlet oxygen and lipid peroxidation Generation of free radical at high oxygen concentration Synergistic antioxidants in biological membranes inhibiting lipid peroxidation Activation transcription factors of antioxidant enzymes Induce the expression of genes encoders for the synthesis of some of the antioxidant enzymes [1]
Beta-carotene	Anti	Yes	
Cryptoxanthin, beta	Anti	Yes	
Lutein-Zeaxanthin	Anti	Yes	
Lycopene<br>Retinol	Anti	Yes	
Alcohol (ethanol)	Pro	Yes	Possible increase in ROS generation and increase of inflammatory processes Induction of OS by oxidation of ethanol to acetaldehyde, which can lead to the production of ROS and RNS, oxidation of nucleic acids and decrease in the activity of antioxidant enzymes. [33]
Total dietary fat	Pro	Yes	Intake of lipids can contribute to oxidative stress through lipid peroxidation [1]
Saturated fat	Pro		Oxidative DNA damage [1]
Avg. daily omega-3 fatty acids+ supplements	Anti	Yes	N-3-PUFAs are involved in antioxidant activity [1]
Avg. daily omega-6 fatty acids + supplements	Pro	Yes	Fatty acids n-6 are pro-inflammatory [1]
Dietary fiber	Anti	Yes	Transported of antioxidants through the intestines and influences the redox through microbiota [2]
Vitamin E as alpha-tocopherol + supplements	Anti	Yes	Fatty acids present in lipoproteins, biological membranes, and tissues, through the elimination of free radicals such as the radical peroxide<br>Protection of the cell membrane, as well as of various subcellular membranes, against the effects of lipid peroxidation Inhibition of lipid peroxidation in biological membranes Protection against the oxidation of LDL-cholesterol Prevention against risk factors or diseases initiated or promoted by ROS [1]
Caffeine	Anti	Yes	Binds copper and Iron to prevent oxidation[16]
Copper + supplements	Anti		Metabolic role in reduction of oxidative stress[8 10]

Iron+ supplements	Pro	Yes	Oxidative DNA damage [1]
Magnesium + supplements	Anti		Required for glutathione production [7]
Selenium + supplements	Anti	Yes	Oxidative DNA damage [1]
Zinc, total + supplements	Anti	Yes	Cofactors of enzymes involved in the endogenous antioxidant system that interrupt cellular oxidative processes [1]
Glutathione, total	Anti	Yes	Prevention of the formation of free radicals, metal chelators [17]
Total Flavonoid	Anti	Sum from the list of Flavonoids below.*	Inhibition of expression, synthesis or activity of pro-oxidant enzymes<br>Induce the expression of genes encoders for the synthesis of some of the antioxidant enzymes [1]
Vitamin C + supplements	Anti	Yes	Antioxidant that scavenges ROS and RNS<br>Prevention of lipid peroxidation<br>Regeneration of _-tocopherol [11]
Niacin+ supplements	Anti		Acts as a hydride ion acceptor or donor in many biological redox reactions. [4]
Riboflavin (Vitamin B2) + supplements	Anti		A coenzyme in numerous redox reactions[6]
Average daily Dietary Folate Equivalents + supplements	Anti	Yes	Improve the endogenous antioxidant system. Also, folic acid increases the ratio of reduced/oxidized glutathione (GSH/GSSG) and reduces protein nitration [12]
Vitamin D + supplements	Anti	Yes	Vitamin D passes through the expression of the erythroid-derived 2 nuclear factor Nfr2, which under ROS production activates the expression of antioxidant enzymes.
Vitamin K as phylloquinone + supplements	Anti		Cofactor in redox modulation of NADPH and Glutathione [13]
HEI-2010 component 12, SoFAAS Cals (Solid Fat, Excess Alcohol & Added Sugars	Pro	No	Composite score that integrates saturated, fat, alcohol and added sugars, all known to be pro antioxidants
Red meat	Pro	Yes	Possible intensification of oxidative stress mediated by iron intake contained in red meat [1]
Brassica vegetables (Greens, cabbage, broccoli, spinach)	Anti	Add the highlighted together as a total estimate of Cruciferous or *Brassica* veget ables vegetable intake	Attempt to distinguish higher intake of vegetables with phytochemicals compared to higher vegetable intake [9,34]
Smoking Status	pro		Exogenous prooxidant: increased oxidative stress and oxidative imbalance in cellular

			tissues The increase of the OS load of inhaled tobacco smoke could increase through the secondary release of oxygen radicals from the inflammatory cells Increase in markers of oxidative stress in blood and tissues [1]
**Medication/Lifestyle Choices from 2nd trimester of pregnancy**			
NSAID Use	Anti	Yes	Regulation of ROS and RNS to reduce inflammation and cell damage[1]
BMI	Pro	Yes	Related to increased ROS markers Leads to redox imbalance and increased lipid peroxidation, which can lead to ROS production [35]
**Biomarkers from 2nd trimester of pregnancy**			
Vitamin D	Anti	No	Vitamin D passes through the expression of the erythroid-derived 2 nuclear factor Nfr2, which under ROS production activates the expression of antioxidant enzymes. [1].
Plasma Folate	Anti	No	Improves the endogenous antioxidant system. Also, folic acid increases the ratio of reduced/oxidized glutathione (GSH/GSSG) and reduces protein nitration [12].

Table S3: Socioeconomic characteristics of the study population.

Characteristic	Full CANDLE Cohort N=1503 mean (SD)/N (%)	Eligible Study Sample N=1322 mean (SD)/N (%)	P value
**Maternal age, y**	26.0 (5.4)	26.3 (5.4)	0.2101
**Maternal race**			
Black	990 (65.9%)	847 (64.1%)	0.3127
**Maternal education**			
High school diploma or GED or less	893 (59.2%)	761 (57.6)	0.8842
**Pre-pregnancy BMI status**			
Overweight/Obese	799 (53.3%)	701 (53.2%)	0.9048
**Maternal marital status**			
Cohabitation	848 (56.4%)	777 (58.8%)	0.2206
**Health insurance status**			
Public	901 (60.0%)	755 (57.1%)	0.1364
**Adjusted household income**[1]			
≥$40k	201 (14.7%)	183 (15.1%)	0.8196
**Prenatal alcohol use (yes)**	121 (8.1%)	115 (8.7%)	0.5795
**Prenatal tobacco use (yes)**	151 (10.1%)	132 (10.0%)	1.0000
**Plasma 25(OH)D, ng/mL**	21.7 (8.5)	22.3 (8.6)	0.0670
**Plasma Folate, ng/mL**	23.6 (12.9)	23.8 (12.9)	0.6565

[1]Adjusted for size of household.

Table S4: Food Items from Food Frequency Questionnaire associated with Oxidative Balance Score (R^2=0.82).

Food Items	Food Group	Beta[1]
Liver	Organ meats	1.08
Oysters	Fish and other seafood	0.76
Tofu	Tofu and meat substitutes	0.65
Spinach	Cruciferous vegetable	0.49
Carrots	Yellow vegetables	0.39
Sweet potato	Yellow vegetables	0.31
Shellfish	Fish and other seafood	0.29
Hot tea	Teas	0.23
Green salad	Salad dressing	0.21
Power bars	Snacks	0.20
Spaghetti with meat sauce	Rice, pasta, mixed, dishes	0.19
Broccoli	Cuciferious vegetable	0.17
Slimfast	Dairy	0.17
Coffee	Coffee	0.16
Vegetable stew	Other vegetables	0.14
Other vegetables	Other vegetables	0.11
Beef	Red meat	0.10
Other fresh fruit	Other fruit	0.09
Tomato juice	Tomatoes	0.09
Real orange juice	Fruit juices	0.08
Milk on cereal	Dairy	0.07
Strawberries, in season	Citrus fruits	0.07
Nuts	Nuts	0.07
Breakfast bars	Snacks	0.06
Ice tea	Teas	0.05
Milk (default 2%)	Dairy	0.05
Meat substitutes	Tofu and meat substitutes	0.04
Other potatoes	Potatoes	0.04
Bagels, English muffins	Bread products	0.04
Cold cereal	Cold breakfast cereal	0.04
Bean soup	Legumes	0.04
Other noodles	Rice, pasta, mixed, dishes	0.04
Yogurt	Dairy	0.04
Oranges or tangerines	Citrus fruits	0.03
Tacos	Mixed meat	0.03
Greens	Cruciferous vegetable	0.03
Vegetable soup	Other vegetables	0.03
Watermelon, in season	Other fruit	0.02
Pork	Red meat	0.02
Water	Water	0.02
Bananas	Other fruit	0.02
Other beans	Legumes	0.02
Apples or pears	Other fruit	0.01
Catsup	Condiments	0.01

Cooked Cereal	Whole grains	0.01
Other soup	Rice, pasta, mixed, dishes	0.01
Chocolate syrup	Chocolate	0.01
Other fish	Fish and other seafood	0.01
Canned fruit	Canned fruit	0.01
Peaches, raw	Other fruit	0.01
White bread	Bread products	0.01
Other real juice	Fruit juices	0.004
Peanut butter	Nuts	0.003
Lunch Meats	Processed meat	0.002
Hi C	Sugar-sweetened beverages	0.0006
Crackers	Snacks	-0.001
Burger rolls	Bread products	-0.004
Salad dressing	Salad dressing	-0.005
Hamburger (cheeseburger)	Red meat	-0.01
Cookies	Sweets and desserts	-0.01
Pumpkin Pie	Sweets and desserts	-0.01
Bacon	Processed meat	-0.011
Butter	Mayonnaise, margarine and butter	-0.014
Sodas	Sugar-sweetened beverages	-0.01
Chocolate candy	Chocolate	-0.01
Cheese	Dairy	-0.01
Other eggs	Egg	-0.02
Biscuits	Bread products	-0.02
Corn bread	Bread products	-0.02
Rice	Rice, pasta, mixed, dishes	-0.02
Margarine	Mayonnaise, margarine and butter	-0.02
Cake	Sweets and desserts	-0.02
Pancakes	Bread products	-0.02
Mayonnaise	Mayonnaise, margarine and butter	-0.02
Kool aid	Sugar-sweetened beverages	-0.02
Other beef dish	Mixed meat	-0.03
Donuts	Sweets and desserts	-0.03
Fried fish	Fried chicken and fish	-0.03
Pizza	Pizza	-0.03
French Fries	French fries	-0.03
Chips	Snacks	-0.04
Ice cream	Dairy	-0.04
Fried chicken	Fried chicken and fish	-0.04
Mac N Cheese	Rice, pasta, mixed, dishes	-0.05
Cole slaw	Cruciferous vegetable	-0.07
Breakfast sausage	Processed meat	-0.07
Other pie	Sweets and desserts	-0.08
Hot dogs	Processed meat	-0.09
Breakfast egg sandwich	Egg	-0.13
Ribs	Red meat	-0.35
Liquor	Alcohol	-7.31
